# Local uterine resection with Bakri balloon placement in placenta accreta spectrum disorders

**DOI:** 10.4274/tjod.galenos.2020.82652

**Published:** 2020-07-29

**Authors:** Emin Üstünyurt

**Affiliations:** 1 University of Health Sciences Turkey, Bursa Yüksek İhtisas Training and Research Hospital, Clinic of Gynecology, Bursa, Turkey

**Keywords:** Conservative technique, placenta accreta, placenta accreta spectrum, placenta percreta, local resection

## Abstract

**Objective:**

Placenta accreta spectrum (PAS) is a potentially life-threatening condition characterized by the abnormal adherence of the placenta to the implantation site. We sought to evaluate the efficacy, surgical feasibility, risks, and advantages of local uterine resection in cases complicated with PAS.

**Materials and Methods:**

This study included 97 patients with PAS, which was confirmed during surgery and by histopathological examination between January 2013 and December 2019. The patients were divided into two groups based on operative approach. The study population (local resection group) consisted of 30 cases in whom total resection of adherent placenta and myometrium was performed, whereas the control group (hysterectomy group) of 67 cesarean hysterectomy cases.

**Results:**

Patients who underwent hysterectomy had significantly more bleeding than the local resection group (1180±160 mL vs 877±484 mL; p=0.002). The mean number of transfused packed red blood cells (pRBCs) was greater in the hysterectomy group (4.5±2.3) than in the local resection group (2.6±3.1; p=0.001). Transfusion rate of four and/or more pRBCs was 67.2% in the hysterectomy group and 33.3% in the local resection group, which indicated a statistically significant difference (p=0.002). Of patients, 29.6% required intensive care unit in the hysterectomy group and 6.7% in the local resection group (p=0.023).

**Conclusion:**

Local resection can be performed safely in selected PAS cases. In these cases, using a standardized protocol in terms of patient selection and surgical procedure will reduce morbidity and mortality.

**PRECIS:** Local uterine resection can be performed safely in selected cases with placenta accreta spectrum disorder.

## Introduction

Placenta accreta spectrum (PAS) is a potentially life-threatening condition characterized by the abnormal adherence of the placenta to the implantation site^(1)^. It has been more than 80 years since PAS was first defined^(2)^. In a review of 18 cases, the condition was accurately defined as the abnormal adherence of the placenta in whole or partially to the uterine wall^(2)^. It has been shown that iatrogenic damage to the endometrial lining and underlying myometrium can be linked to PAS in subsequent pregnancies^(3,4,5)^. Risk factors for PAS primarily include previous uterine surgery. Epidemiological data indicate that previous cesarean section delivery history and placenta previa diagnosis are major risk factors, and others include advanced maternal age, smoking, dilatation and curettage, and uterine artery embolization^(5,6,7)^. The prevalence of PAS in the general population varies according to local and regional cesarean delivery rates^(8)^. The overall prevalence of PAS has been reported to be 5.2 per 1000 pregnancies, although rates as high as 12.2 per 1000 pregnancies have been reported^(8)^.

Planned cesarean hysterectomy leaving the placenta *in situ* is currently the recommended approach by the American College of Obstetricians and Gynecologists^(9)^. Similarly, the Royal College of Obstetricians and Gynecologists state that cesarean hysterectomy is the preferable approach in PAS^(10)^. However, conservative approach has been described in the literature to preserve future fertility and avoid hysterectomy-related complications such as massive transfusion, coagulopathy, and operative injury^(11)^. The conservative approach defines various surgical techniques that aim to avoid hysterectomy. Four types of conservative management have been described: Extirpative approach, leaving the placenta *in situ*, one-step surgery, and the triple-P procedure^(12)^. The one-step surgery is defined as the resection of the entire adherent placenta with the underlying myometrium^(13)^. The main advantage of this procedure is the relatively lower blood loss compared with manual extirpation as the technique basically consists of controlled surgical en-block excision of the adherent placenta. In addition, as no placental tissue remains following the procedure, persistent risk of bleeding or infection is minimal. The technique has a high success rate, but only few studies with small sample sizes have been reported so far^(13,14,15)^. Therefore, in this study, we sought to evaluate the efficacy, surgical feasibility, risks, and advantages of local uterine resection in the case of PAS.

## Materials and Methods

**Figure 1:**
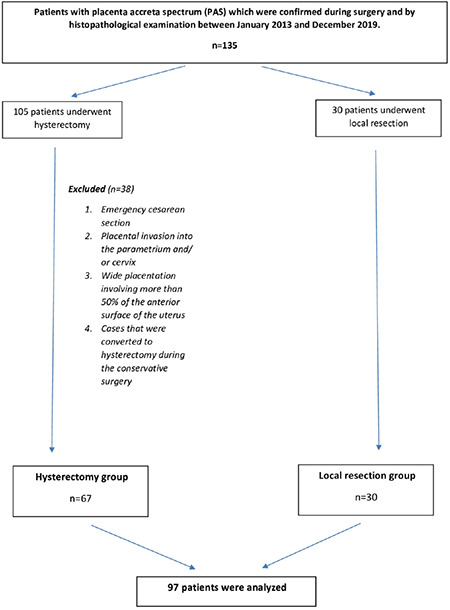
Flowchart of the study PAS: Placenta accreta spectrum

This study is a retrospective analysis of cases that were followed up or referred with a diagnosis of PAS at the University of Health Sciences Turkey, Bursa Yüksek İhtisas Training and Research Hospital, Clinic of Gynecology, which is a tertiary referral medical center with approximately 13,000 deliveries each year. The study was reviewed by the Ethics Committee of University of Health Sciences Turkey, Bursa Yüksek İhtisas Training and Research Hospital, (approval number: 2011-KAEK-25 2019/08-14) and was conducted in accordance with the ethical standards described in an appropriate version of the 1975 Declaration of Helsinki, as revised in 2000. After the approval of the ethics committee, medical records of 135 patients with PAS, which were confirmed during surgery and by histopathological examination between January 2013 and December 2019, were evaluated. Patients who were not diagnosed during the antenatal period and emergency operations and who underwent cesarean hysterectomy were excluded if they did not meet the inclusion criteria of conservative surgery (cases with invasion into the parametrium and/or cervix and invasion of more than 50% of the anterior surface of the uterus). Cases that were converted to hysterectomy during conservative surgery were also excluded from the study. Finally, 97 patients were included (Figure 1), and the patients were recruited into two groups based on operative approach. The study population (local resection group) consisted of cases in whom total resection of adherent placenta and myometrium was performed, whereas the control group (hysterectomy group) of cesarean hysterectomy cases.

The diagnosis of PAS was suspected when transvaginal sonography combined with Doppler studies revealed placenta previa with additional sonographic findings^(16)^. At our institution, hysterectomy is preferred primarily in PAS cases. However, if sonographic and intraoperative findings are appropriate in patients who prefer the preservation of the uterus, uterine sparing surgery is performed. Conservative surgery is not performed in cases with invasion into the parametrium and/or cervix and invasion of more than 50% of the anterior surface of the uterus.

All patients prenatally diagnosed with PAS were fully informed of benefits and risks of the surgical procedures. Signed informed consent was obtained from the patients before the surgery. All operations were performed by experienced surgeons using a standard technique.

All patients were placed in the dorsal lithotomy position during the surgeries. A vertical midline skin incision was made in both hysterectomy and conservative operations. The surgical technique for classical hysterectomy can be briefed as a fundal uterine incision devoid of placental attachment, delivery of the baby, rapid closure of the incision leaving the placenta in situ, and performance of hysterectomy.

As for local resection, the first step is the creation of a plane between the placenta and bladder. Advanced bipolar device (LigaSure, Covidien, Boulder, CO, USA) was used to dissect the vesicouterine space. Dissection was performed until the cervical internal ostium level. The second step was to make a transverse incision to the uterus close but not through the placental insertion site and delivery of the fetus. The next step was the resection of all invaded myometrial tissue and adherent placenta in one piece. Following surgical procedures for hemostasis, the myometrium was sutured in two planes. Intrauterine balloon tamponade (Bakri Postpartum Balloon, Cook Medical, Spencer, IN, USA) and pelvic drains were used in all cases for at least 24 hours.

The following data were obtained from the patients’ records: age, parity, body mass index, uterine surgery history (including cesarean, myomectomy, or dilatation and curettage), gestational age at delivery, perioperative and postoperative bleeding, and histopathological specimen diagnosis. The following data were obtained from the operative note: operative time, estimated blood loss, number of packed red blood cell (pRBC) transfusions, and presence of intraoperative complications. Maternal postoperative complications, neonatal birthweight, and outcomes were also retrieved from the patients’ charts.

### Statistical Analysis

Statistical analysis was performed using Statistical Package for the Social Sciences version 18 (Chicago, IL, USA). Student’s t-test was performed for parametric variables between groups that distribute normally. Mann-Whitney U test was performed for parametric variables without normal distribution, and the chi-square test for nonparametric variables between groups. Multivariate logistic regression analysis was used to investigate the factors affecting transfusion requirement and intensive care unit (ICU) admission. The data were adjusted for other confounders such as age, gravidity, parity, previous cesarean delivery, curettage, and myomectomy. A p-value less than 0.05 was considered significant.

## Results

Clinical and demographic features of the study population are depicted in Table 1. Patients in the hysterectomy group were older those in the local resection group (33.2±4.7 vs 31.1±5.4, p=0.048). Parity >3 was more frequent in patients who had undergone hysterectomy (28.4% vs 13.3%; p=0.036). A previous cesarean delivery history was more frequent in patients in the hysterectomy group (100% vs 76.6%, p<0.001), whereas a previous myomectomy history was more frequent in the local resection group (36.7% vs 10.4%, p=0.002). The rate of previous dilatation and curettage was similar in both groups (p=0.575), and there were no significant differences in histopathologic diagnoses between the groups (p=0.485).

Perioperative data and maternal and neonatal outcomes are presented in Table 2. There were no statistically significant differences between the groups regarding gestational age at delivery and preoperative and postoperative hemoglobin and fibrinogen levels. Likewise, the duration of operation was similar in both groups (102±12 min for the local resection group vs 99±15 min for the hysterectomy group; p=0.255). Patients in whom hysterectomy was performed had significantly more bleeding than the local resection group (1180±160 mL vs 877±484 mL; p=0.002). The mean number of transfused pRBCs was greater in the hysterectomy group (4.5±2.3) than in the local resection group (2.6±3.1; p=0.001).

Intraoperative and postoperative complications occurred at a higher rate in patients in the hysterectomy group than those in the local resection group. Four and more pRBCs transfusion rates were 67.2% in the hysterectomy group and 33.3% in the local resection group, which indicated a statistically significant difference (p=0.002). In the hysterectomy group, two patients developed disseminated intravascular coagulation, and one developed acute renal failure. The rate of bladder injury in the hysterectomy group was higher than the local resection group, but that did not reach statistical significance [15 (22.4%) vs 3 (10.0%); p=0.147, respectively). The rate of ICU admission was higher in the hysterectomy group than the local resection group. Of patients, 29.6% required ICU in the hysterectomy group and 6.7% in the local resection group (p=0.023). Neonatal outcomes in terms of birthweight, 5-min Apgar scores, and neonatal ICU admission were similar in both groups.

In the multivariate logistic regression analysis model, after adjusting for relevant confounding factors (age, gravidity, parity, previous cesarean delivery, curettage, and myomectomy), hysterectomy was an independent risk factor for four or more pRBC transfusion requirement (odds ratio (OR): 9.442, 95% confidence interval (CI): 2.072-43.026, p=0.004) and ICU admission (OR: 10.092, 95% CI: 2.363-42.376, p=0.007; Tables 3, 4).

## Discussion

PAS frequency is increasing rapidly because of the increase in cesarean section rates today. Planned cesarean section hysterectomy has been considered the main treatment option for these cases, consistent with the American College of Obstetricians and Gynecologists recommendations^(17)^. However, many patients are young and with low parity, and uterine conservative approaches should be considered in these patients with a further fertility desire. Palacios-Jaraquemada et al.^(18)^ reported that the uterus could be preserved in 80% of patients without causing additional morbidity in the study where they examined 248 PAS cases. In a prospective study involving 20 PAS cases, only 1 (5%) patient required hysterectomy when performing the triple-P procedure, which includes removal of the fetus through a separate incision over the placental site, ligation of bilateral uterine artery, and excision of the relevant myometrial region without separating the placenta^(19)^.

Both definitive and conservative surgical approaches of patients with PAS are associated with increased risk of maternal morbidity and mortality. The important point here is that uterine-sparing interventions should not pose an extra risk in patients in terms of complications such as bleeding and adjacent organ injuries compared with cesarean section hysterectomy. According to published data, transfusion is required in up to 90% of these patients^(20)^. Estimated blood losses related to cesarean hysterectomy in PAS cases are 3000-5000 mL^(21,22)^. In a meta-analysis, which examined 29 studies including 7001 PAS cases, the frequency of transfusion-requiring hemorrhage was 46.9%^(23)^. In the publications related to conservative PAS surgery, the average amount of bleeding is 1630 cc, and the average pRBC transfusion is 3.9 units^(24)^. Bladder injury, reported as approximately 10% to 30%, is another common complication in PAS cases^(25,26)^. In a study involving 65 PAS cases, urinary tract injury was detected in 31.4% (16/51) and 14% (2/14) of those who underwent cesarean hysterectomy and conservative surgery, respectively^(27)^. Here, bladder injury occurred 2.5 times more than ureter injury^(27)^.

In this study, we investigated whether conservative surgery is acceptable in selected PAS cases. The findings indicate that local resection causes less morbidity in selected PAS cases compared with hysterectomy. Local resection was associated with reduced bleeding, transfusion requirement, and ICU admission rate compared with hysterectomy. After adjusting for relevant confounding factors, hysterectomy was an independent risk factor for four or more pRBC transfusion requirements and ICU admission. Additionally, the rate of bladder injury was higher in patients who underwent hysterectomy, although this did not reach statistically significant difference. Our findings are consistent with the results of publications related to conservative PAS surgery^(18,24,28,29,30)^. In many studies, it is reported that bleeding and bladder injuries occur during vesicouterine space dissection, which is a common step in both local resection and hysterectomy^(24,27)^. Despite these data, some explanations can be made about why these complications occur less frequently in conservative surgery. Many factors such as placental location and invasion depth, time of diagnosis, transfusion capabilities of the center where the operation is performed, and the surgeon’s experiences affect the morbidity and mortality in PAS cases. Many clinics, including us, begin performing conservative procedures after gaining enough experience in PAS surgery^(18)^. Another point is that conservative surgery may have been preferred in relatively low-grade PAS cases. In addition, the fact that hysterectomy is an extensive surgical procedure than local resection may also contribute to the increase in morbidity in hysterectomy cases.

In our view, some important points should be considered to perform a conservative surgery in PAS cases without increasing morbidity and mortality. Conservative surgery should be preferred only in selected PAS cases. Thus, the cases should be evaluated in detail with ultrasonography and other imaging modalities before operation. Each surgeon should determine the conservative surgical acceptance criteria according to his or her own experience and skills. The most important step of surgery is the dissection of the vesicouterine space where major bleeding occurs due to neovascularization. We believe that performing this step initially before delivery of the fetus and using advanced bipolar device can significantly decrease the amount of bleeding. During the bladder dissection or at any operation stage, any bleeding that cannot be controlled is encountered, and definitive surgical procedure should not be delayed. It has been reported that balloon tamponade reduces blood loss and transfusion amounts, although it has a higher failure rate in the presence of PAS^(31,32,33)^ (AJOG). We believe that the routine use of Bakri in these cases is beneficial in terms of reducing and following up bleeding in the postpartum period. In our institution, we do not perform prophylactic hypogastric artery ligation. We believe that this approach is time consuming and not effective. It is performed only if the previous steps such as balloon tamponade or hysterectomy have failed to control the bleeding.

### Study Limitations

This was a retrospective study with susceptibility to selected bias. The subjective criteria of the surgeon may have influenced the selection of the surgical type of the patients. The severity of PAS cases was basically evaluated according to ultrasonography and surgery notes. Since it was not specified in detail in some surgical and sonography records, the hysterectomy group may have included more serious cases than the local resection group.

One of the strengths of this study is that unlike other published studies, two comparable groups were provided by excluding hysterectomy cases that did not meet the criteria for conservative surgery. Additional strengths include relatively larger sample size and standard surgical technique application in operations.

## Conclusion

Our findings suggest that local resection can be performed safely in selected PAS cases. In these cases, using a standardized protocol in terms of patient selection and surgical procedure will reduce morbidity and mortality.

## Figures and Tables

**Table 1:**
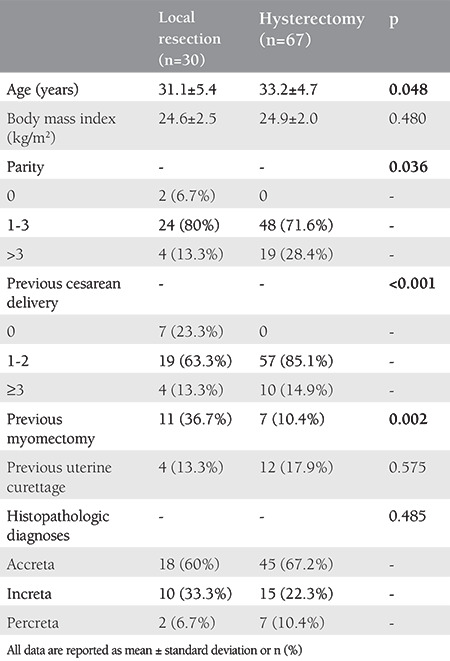
Characteristics of the study population

**Table 2:**
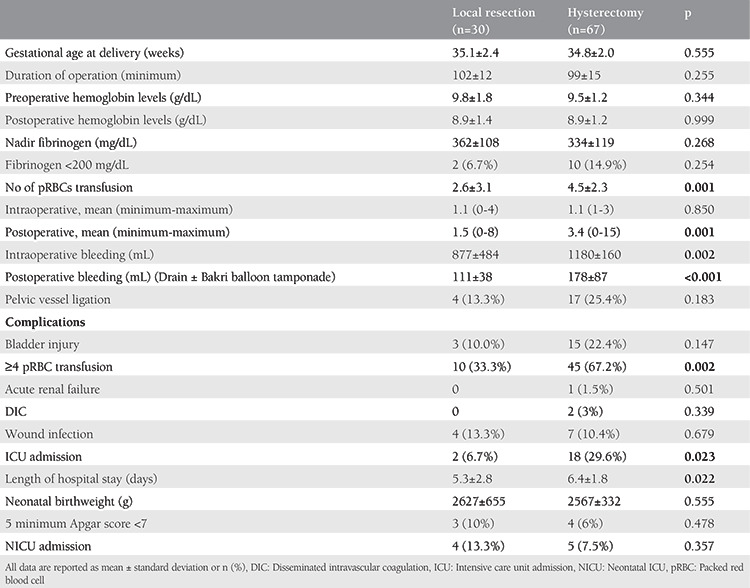
Perioperative data and maternal and neonatal outcomes

**Table 3:**
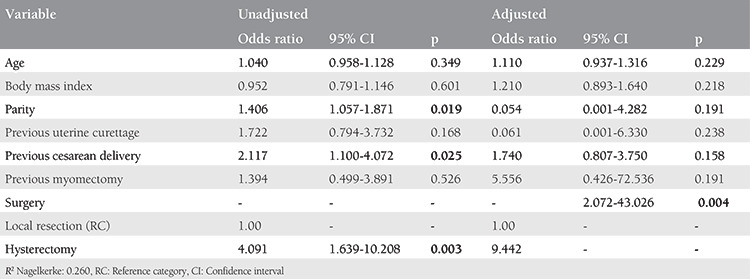
Logistic regression analysis for factors affecting four or more packed red blood cell transfusion requirement

**Table 4:**
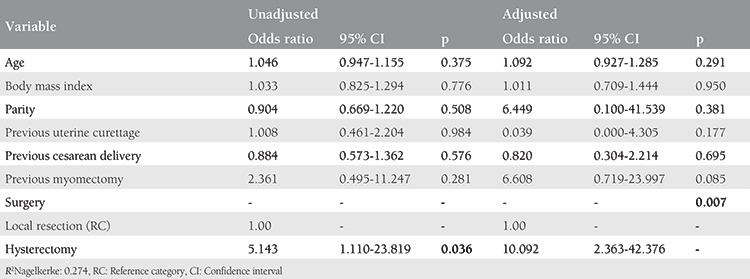
Logistic regression analysis for the factors affecting intensive care unit admission

## References

[1] D’antonio F, Iacovella C, Bhide A (2013). Prenatal identification of invasive placentation using ultrasound: Systematic review and meta‐analysis. Ultrasound Obstet Gynecol.

[2] Irving F (1937). A study of placenta accreta. Surg Gynecol Obstet.

[3] Jauniaux E, Jurkovic D (2012). Placenta accreta: pathogenesis of a 20th century iatrogenic uterine disease. Placenta.

[4] Bowman ZS, Eller AG, Bardsley TR, Greene T, Varner MW, Silver RM (2014). Risk factors for placenta accreta: A large prospective cohort. Am J Perinatol.

[5] Jauniaux E, Collins SL, Jurkovic D, Burton GJ (2016). Accreta placentation: a systematic review of prenatal ultrasound imaging and grading of villous invasiveness. Am J Obstet Gynecol.

[6] Fox KA, Shamshirsaz AA, Carusi D, Secord AA, Lee P, Turan OM, et al (2015). Conservative management of morbidly adherent placenta: expert review. Am J Obstet Gynecol.

[7] Jauniaux E, Grønbeck L, Bunce C, Langhoff-Roos J, Collins SL (2019). Epidemiology of placenta previa accreta: a systematic review and meta-analysis. BMJ Open.

[8] Cresswell JA, Ronsmans C, Calvert C, Filippi V (2013). Prevalence of placenta praevia by world region: a systematic review and meta-analysis. Trop Med Int Health.

[9] No OCC (2018). 7: placenta accreta spectrum. Obstet Gynecol.

[10] Jauniaux E, Alfirevic Z, Bhide A, Belfort M, Burton G, Dornan S, et al (2019). Placenta praevia and placenta accreta: diagnosis and management. Green-top Guideline No. 27a. BJOG.

[11] Sentilhes L, Kayem G, Silver RM (2018). Conservative management of placenta accreta spectrum. Clin Obstet Gynecol.

[12] Sentilhes L, Kayem G, Chandraharan E, Palacios‐Jaraquemada J, Jauniaux E, Diagnosis FPA, et al (2018). FIGO consensus guidelines on placenta accreta spectrum disorders: conservative management. Int J Gynaecol Obstet.

[13] Palacios Jaraquemada JM, Pesaresi M, Nassif JC, Hermosid S (2004). Anterior placenta percreta: surgical approach, hemostasis and uterine repair. Acta Obstet Gynecol Scand.

[14] Karaman E, Kolusarı A, Çetin O, Çim N, Alkış İ, Yıldızhan R, et al (2017). Local resection may be a strong alternative to cesarean hysterectomy in conservative surgical management of placenta percreta: experiences from a tertiary hospital. J Matern Fetal Neonatal Med.

[15] Zhao X, Tao Y, Du Y, Zhao L, Liu C, Zhou Y, et al (2018). The application of uterine wall local resection and reconstruction to preserve the uterus for the management of morbidly adherent placenta: case series. Taiwan J Obstet Gynecol.

[16] Bowman ZS, Eller AG, Kennedy AM, Richards DS, Winter III TC, Woodward PJ, et al (2014). Accuracy of ultrasound for the prediction of placenta accreta. Am J Obstet Gynecol.

[17] Practice CoO (2012). Committee opinion no. 529: placenta accreta. Obstet Gynecol.

[18] Palacios-Jaraquemada JM, Fiorillo A, Hamer J, Martínez M, Bruno C (2020). Placenta accreta spectrum: a hysterectomy can be prevented in almost 80% of cases using a resective-reconstructive technique. J Matern Fetal Neonatal Med.

[19] Abo-Elroose AA-E, Ahmed MR, Shaaban MM, Ghoneim HM, Mohamed TY (2019). Triple P with T-shaped lower segment suture; an effective novel alternative to hysterectomy in morbidly adherent anterior placenta previa. J Matern Fetal Neonatal Med.

[20] O’Brien JM, Barton JR, Donaldson ES (1996). The management of placenta percreta: conservative and operative strategies. Am J Obstet Gynecol.

[21] Kayem G, Deneux‐Tharaux C, Sentilhes L (2013). PACCRETA: Clinical situations at high risk of placenta ACCRETA/percreta: impact of diagnostic methods and management on maternal morbidity. Acta Obstet Gynecol Scand.

[22] Bateman BT, Mhyre JM, Callaghan WM, Kuklina EV (2012). Peripartum hysterectomy in the United States: nationwide 14 year experience. Am J Obstet Gynecol.

[23] Jauniaux E, Bunce C, Grønbeck L, Langhoff-Roos J (2019). Prevalence and main outcomes of placenta accreta spectrum: a systematic review and metaanalysis. Am J Obstet Gynecol.

[24] Nieto-Calvache AJ, Zambrano MA, Herrera NA, Usma A, Bryon AM, Benavides Calvache JP, et al (2019). Resective-reconstructive treatment of abnormally invasive placenta: Inter Institutional Collaboration by telemedicine (eHealth). J Matern Fetal Neonatal Med.

[25] Tam Tam KB, Dozier J, Martin JR JN (2012). Approaches to reduce urinary tract injury during management of placenta accreta, increta, and percreta: a systematic review. J Matern Fetal Neonatal Med.

[26] Norris BL, Everaerts W, Posma E, Murphy DG, Umstad MP, Costello AJ, et al (2016). The urologist’s role in multidisciplinary management of placenta percreta. BJU Int.

[27] Nieto-Calvache AJ, López-Girón MC, Messa-Bryon A, Ceballos- Posada ML, Duque-Galán M, Ríos-Posada JGd, et al (2019). Urinary tract injuries during treatment of patients with morbidly adherent placenta. J Matern Fetal Neonatal Med.

[28] Peng X, Chen D, Xu J, Liu X, You Y, Peng B (2019). Parallel transverse uterine incisions, a novel approach for managing heavy hemorrhage and preserving the uterus: A retrospective cohort study for patients with anterior placenta previa and accreta. Medicine (Baltimore).

[29] Cırpan T, Akdemir A, Okmen F, Hortu I, Ekici H, Imamoglu M (2019). Effectiveness of segmental resection technique in the treatment of placenta accreta spectrum. J Matern Fetal Neonatal Med.

[30] Polat I, Yücel B, Gedikbasi A, Aslan H, Fendal A (2017). The effectiveness of double incision technique in uterus preserving surgery for placenta percreta. BMC Pregnancy Childbirth.

[31] Pala Ş, Atilgan R, Başpınar M, Kavak EC, Yavuzkır Ş, Akyol A, et al (2018). Comparison of results of Bakri balloon tamponade and caesarean hysterectomy in management of placenta accreta and increta: a retrospective study. J Obstet Gynaecol.

[32] Mathur M, Ng QJ, Tagore S (2018). Use of Bakri balloon tamponade (BBT) for conservative management of postpartum haemorrhage: a tertiary referral centre case series. J Obstet Gynaecol.

[33] Maher MA, Abdelaziz A (2017). Comparison between two management protocols for postpartum hemorrhage during cesarean section in placenta previa: Balloon protocol versus non‐balloon protocol. J Obstet Gynaecol Res.

